# Dexamethasone inhibits the Nox-dependent ROS production *via *suppression of MKP-1-dependent MAPK pathways in activated microglia

**DOI:** 10.1186/1471-2202-12-49

**Published:** 2011-05-26

**Authors:** Yingqian Huo, Parakalan Rangarajan, Eng-Ang Ling, S Thameem Dheen

**Affiliations:** 1 Department of Anatomy Yong Loo Lin school of Medicine National University of Singapore, 117597, Singapore

**Keywords:** microglia, Nox-2, MAPKs, ROS, dexamethasone

## Abstract

**Background:**

Nox-2 (also known as gp91*phox*), a subunit component of NADPH oxidases, generates reactive oxygen species (ROS). Nox-dependent ROS generation and nitric oxide (NO) release by microglia have been implicated in a variety of diseases in the central nervous system. Dexamethasone (Dex) has been shown to suppress the ROS production, NO release and inflammatory reaction of activated microglial cells. However, the underlying mechanisms remain unclear.

**Results:**

The present study showed that the increased ROS production and NO release in activated BV-2 microglial cells by LPS were associated with increased expression of Nox-2 and iNOS. Dex suppressed the upregulation of Nox-2 and iNOS, as well as the subsequent ROS production and NO synthesis in activated BV-2 cells. This inhibition caused by Dex appeared to be mediated by upregulation of MAPK phosphatase-1 (MKP-1), which antagonizes the activity of mitogen-activated protein kinases (MAPKs). Dex induced-suppression of Nox-2 and -upregulation of MKP-1 was also evident in the activated microglia from corpus callosum of postnatal rat brains. The overexpression of MKP-1 or inhibition of MAPKs (by specific inhibitors of JNK and p38 MAPKs), were found to downregulate the expression of Nox-2 and iNOS and thereby inhibit the synthesis of ROS and NO in activated BV-2 cells. Moreover, Dex was unable to suppress the LPS-induced synthesis of ROS and NO in BV-2 cells transfected with MKP-1 siRNA. On the other hand, knockdown of Nox-2 in BV-2 cells suppressed the LPS-induced ROS production and NO release.

**Conclusion:**

In conclusion, it is suggested that downregulation of Nox-2 and overexpression of MKP-1 that regulate ROS and NO may form the potential therapeutic strategy for the treatment of neuroinflammation in neurodegenerative diseases.

## Background

An inflammatory process in the central nervous system (CNS) is considered to be a prominent feature in a number of neurodegenerative diseases and is mediated by the activated microglia, the resident immune cells of the CNS. The microglia normally respond to neuronal damage and remove the damaged cells by phagocytosis [1]. The chronic activation of these cells appears to cause neuronal damage through enhanced release of potentially cytotoxic molecules such as proinflammatory cytokines including tumor necrosis factor-α (TNF-α) and interleukin-1β (IL-1β), nitric oxide (NO), reactive oxygen intermediates, proteinases and complement proteins [2-5]. Moreover, microglia-derived free radicals as well as the reactive reaction products, hydrogen peroxide and peroxynitrite, have the potential to harm cells and have been implicated in contributing to oxidative damage and neurodegeneration in neurological diseases [6,7]. Therefore, suppression of microglia-mediated inflammation has been considered as an important strategy in neurodegenerative disease therapy.

Nicotinamide adenine dinuceotide phosphate (NADPH) oxidase, a multi-component enzyme complex is an important source of reactive oxygen species (ROS) in respiratory oxidative stress and intracellular signaling pathways [8,9]. NADPH oxidase has been shown to be involved in the innate immune response by killing microbes through generation of ROS. Nox family is a subunit component of NADPH oxidases that generate superoxide and other downstream ROS. Among them, Nox-2 (also known as gp91*phox*), is expressed in the neuron and glial cells including microglia [10] and regulates NADPH oxidase activities by stabilizing p22phox (also a subunit of NADPH) to form its membrane component. Nox-dependent ROS generation has been shown to regulate the production of cytokines and chemokines as well as other proinflammatory molecules [11,12]. Due to its expression in microglia and ability to generate large amounts of ROS, Nox protein is thus considered to be critical for ROS induction in activated microglia [10].

Recent studies have demonstrated that activated microglia are important sources of ROS and reactive nitrogen species (RNS) in the brain and involved in neuroinflammation processes in neurodegenerative diseases [13,14]. Abundant existence of ROS has been found to be associated with neurodegenerative disorders such as Parkinson's disease and Alzheimer's disease [15-17]. It has also been reported that large amount of nitric oxide (NO) production catalyzed by inducible NO synthase (iNOS) may contribute to cellular damage in the CNS [18,19]. Furthermore, attempts made to restrict the oxidative stress have been proven to be of beneficial to patients with neurodegenerative disorders. These pieces of evidence indicate that limiting oxidative stress is an important step in controlling neurodegenerative diseases.

Glucocorticoids (GC), the endogenous immunosuppressors for the innate immune response and the subsequent inflammatory reaction, have been shown to enhance the survival of several phagocytic cells by suppressing intracellular ROS production and inhibiting ROS-induced apoptosis [20]. Dexamethasone (Dex), a synthetic glucocorticoid, has also been demonstrated to inhibit iNOS expression and NO production in LPS-induced macrophages [21].

Mitogen activated protein kinase (MAPK) pathways are important signaling cascades that mediate several cellular functions by relaying extracellular signals to key intracellular molecules [22]. It has been previously shown that the induction of the MAPK pathways leads to microglial activation by transcription of TNFα, Cox-2 and MCP-1 [23,24]. Three particular MAPK pathways - p38, Jun N-terminal kinase (JNK) and extracellular signal-regulated kinase (ERK) pathways have been shown to be regulated by GC *via *activating the expression of MAPK Phosphatase-1 (MKP-1) [25]. We have demonstrated that Dex suppresses microglia-involved inflammation by inhibiting production of monocyte chemoattractant protein-1 (MCP-1), a chemokine and subsequent migration of microglia *via *MAPK phosphatase-1 (MKP-1) dependent suppression of p38 and JNK MAPK pathways in activated microglia [26]. However the effect of Dex on ROS production and its possible regulatory mechanism in activated microglia remains to be demonstrated. In this study, we demonstrate that Dex inhibits ROS production by downregulating the expression of Nox-2 and by MKP-1 dependent suppression of p38 and JNK MAPK pathways. These results reveal new mechanisms by which Dex attenuates ROS which may be helpful in developing therapeutic strategies for minimizing oxidative stress mediated neurodegeneration in CNS diseases.

## Results

### LPS alters the ROS production and Nox-2 protein expression in BV-2 microglial cells in a dose- and time-dependent manner

**Figure 1:**
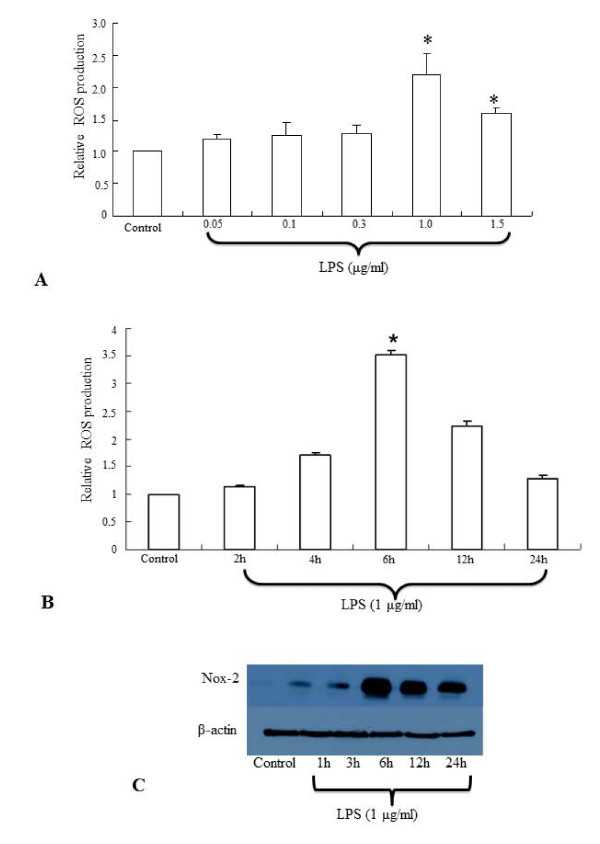
**A-C. Effect of LPS on the ROS production and Nox-2 protein expression in BV-2 microglial cells **. Flow cytometry hitogram shows that ROS production is significantly increased in BV-2 microglial cells treated with LPS at the concentration of 1.0-1.5 μg/ml for 6 h (**A**). Time course analysis reveals that maximum ROS production occurs in BV-2 cells treated with LPS for 6 h (**B**). Western blot analysis shows that Nox-2 protein expression is increased maximally in BV-2 cells treated with LPS at 1 μg/ml for 6 h (**C**). The data represent the mean ± SD (n = 3); * *p *< 0.05

ROS production in the BV-2 microglial cells treated with different concentrations of LPS (0.05, 0.1 and 0.3, 1, 1.5 μg/ml) was estimated in order to choose the optimum dose of LPS for further studies. Although the ROS production in BV-2 cells appeared to be induced by all the concentrations of LPS used, a significant increase in ROS production was observed in BV-2 cells treated with 1-1.5 μg/ml of LPS (Figure. 1A). However, the significant increase in ROS production was found to be declined in cells when the LPS concentration was increased to 1.5 μg/ml. Subsequently, the BV-2 microglial cells were treated with 1 μg/ml of LPS for different time points (2, 4, 6, 12 and 24 h). The ROS production in LPS-activated BV-2 cells was progressively increased with time, peaked at 6 h and declined thereafter (Figure. 1B). Further, the protein expression of Nox-2 was found to be increased maximally in BV-2 cells treated with LPS at 1 μg/ml for 6 h (Figure. 1C). Thus we chose to treat BV-2 cells with LPS at 1 μg/ml for 6 h for the rest of the studies.

### Dexamethasone suppresses the LPS-induced Nox-2 expression in microglia *in vivo *and *in vitro*

**Figure 2:**
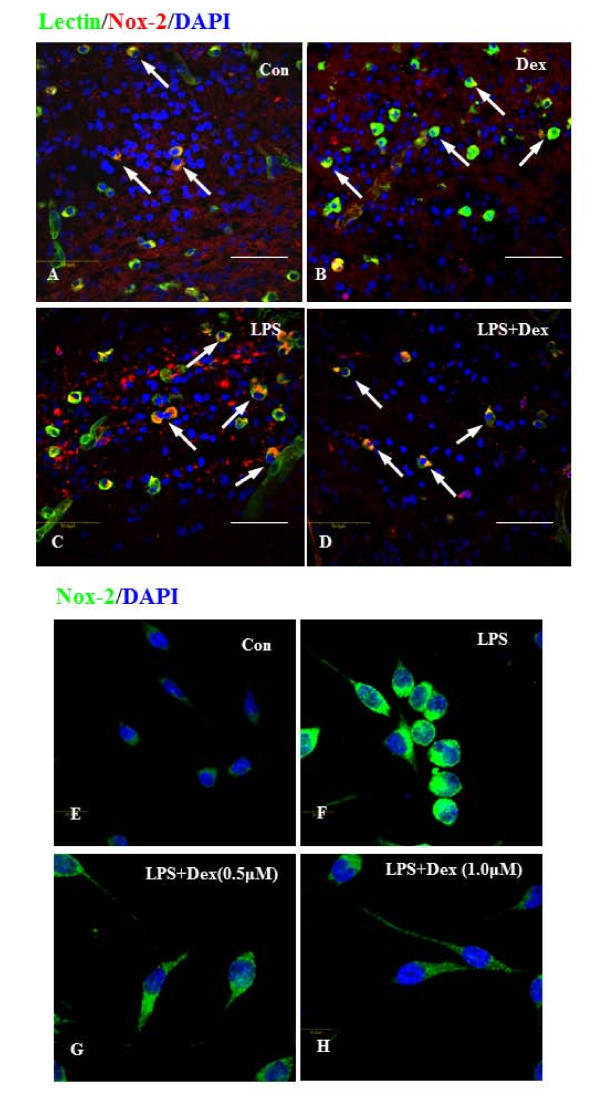
**A-H. Confocal images showing the suppressive effect of Dex on Nox-2 expression in LPS-treated microglia *in vivo *and *in vitro***. Colocalization of lectin (green), Nox-2 (red) and DAPI (blue) was detected in corpus callosum region of brain sections from 3d postnatal rats with or without LPS or LPS+Dex injection (**A-D**). The incidence of Nox-2-positive microglial cells (arrows) was increased in the brain of LPS-injected rats (**C**) compared to control (**A**) and to Dex alone (**B**)and the frequency of Nox-2-positive cells substantially decreased after Dex treatment (**D**). Double labeling was also carried out between Nox-2 (green) and DAPI (blue) in BV-2 cells treated with LPS for 6 h without (**F**) or with Dex (**G**, 0.5 &**H**, 1 μM) *in vitro*. The Nox-2 immunoreactivity appears to be increased in cells treated with LPS (**F**), compared to control (**E**). However, Dex decreased the intensity of immunoreactivity in a dose dependent manner in cells treated with LPS (**G, H**).

Expression of Nox-2 was examined immunohistochemically both *in vivo *and *in vitro*. In normal and Dex treated 3d postnatal rat pups, a considerable number of lectin-positive microglial cells were found to be distributed in the corpus callosum of the brain, and majority of them were colocalized with Nox-2 (Figure. 2AB). The frequency of these cells was markedly increased in the brain of pups which received LPS injection (Figure. 2C); however, injection of Dex decreased the incidence of these cells (Figure. 2D). *In vitro*, the expression of Nox-2 was greatly increased in majority of the BV-2 cells exposed to LPS for 6 h (Figure. 2F), compared to untreated cells (Figure. 2E). Following treatment of Dex at 0.5 μM (Figure. 2G) and 1 μM (Figure. 2H), the Nox-2 immunoreactivity was noticeably decreased in LPS-activated BV-2 cells. The size of activated BV-2 cells appeared to be increased and the size was reduced with the addition of Dex (Figure. 2E-H).

### Dexamethasone and inhibitors of p38 and JNK MAPK pathways suppress the ROS production in activated BV-2 cells

**Figure 3:**
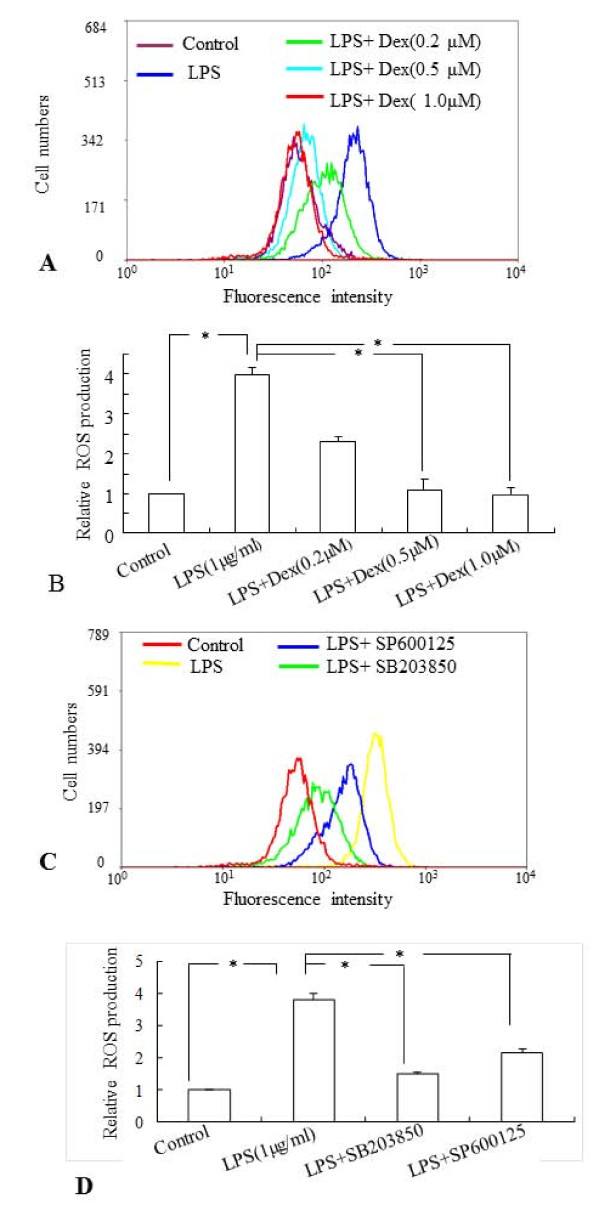
**A-D The flow cytometric and quantitative analyses showing the suppressive effects of Dex and inhibitors of MAPKs on ROS production in BV-2 cells**. LPS induced ROS generation significantly in BV2 cells compared to control and preincubation of Dex suppressed the induction of ROS production in a dose-dependent manner (0.2, 0.5 and 1.0 μM) (**A, B**). Similarly, inhibitors of p38 (SB203580) and JNK (SP600125) suppressed the ROS generation in LPS stimulated BV-2 cells (**C, D**).Quantitative analysis of ROS production in BV-2 cells subjected to different treatments is shown in **B **and **D**. Panels **A **and **C **represent the results of flow cytometric analysis of DCF fluorescence intensity from one sample of each group obtained from three independent experiments performed. The data represent the mean ± SD (n = 3); * *p *< 0.05.

The effect of Dex on ROS production in LPS-treated BV-2 cells was examined by flow cytometry using CM-H2DCFDA as the fluorescent probe. The fluorescence intensity represents the amount of ROS production in the cells. Incubation with LPS (1 μg/ml) for 6 h significantly increased the ROS production in BV-2 cells, when compared to that in untreated cells (Figure. 3A,B). However, pretreatment with Dex at different concentrations (0.2 μM, 0.5 μM and 1.0 μM), 30 min prior to incubation with LPS prevented the increase of ROS production significantly in BV-2 cells (Figure. 3A, B). The inhibitory effect on ROS production in activated BV-2 cells was augmented with the increase in Dex concentrations. On the other hand, Dex alone at different concentrations did not alter ROS production in BV-2 cells significantly, when compared with that in the control (data not shown).

To examine whether the MAPK signaling pathways are involved in the induction of ROS production in activated BV-2 cells, the cells were incubated with the inhibitors of p38 (SB203580) and JNK (SP600125) MAPKs, 30 min prior to the addition of LPS. Flow cytometric and quantitative analysis revealed that the ROS production was significantly decreased in the LPS-stimulated BV-2 cells exposed to either p38 or JNK inhibitors (Figure. 3C,D).

### Dexamethasone and inhibitors of p38 and JNK MAPK pathways suppress iNOS expression in microglial cells treated with LPS

**Figure 4:**
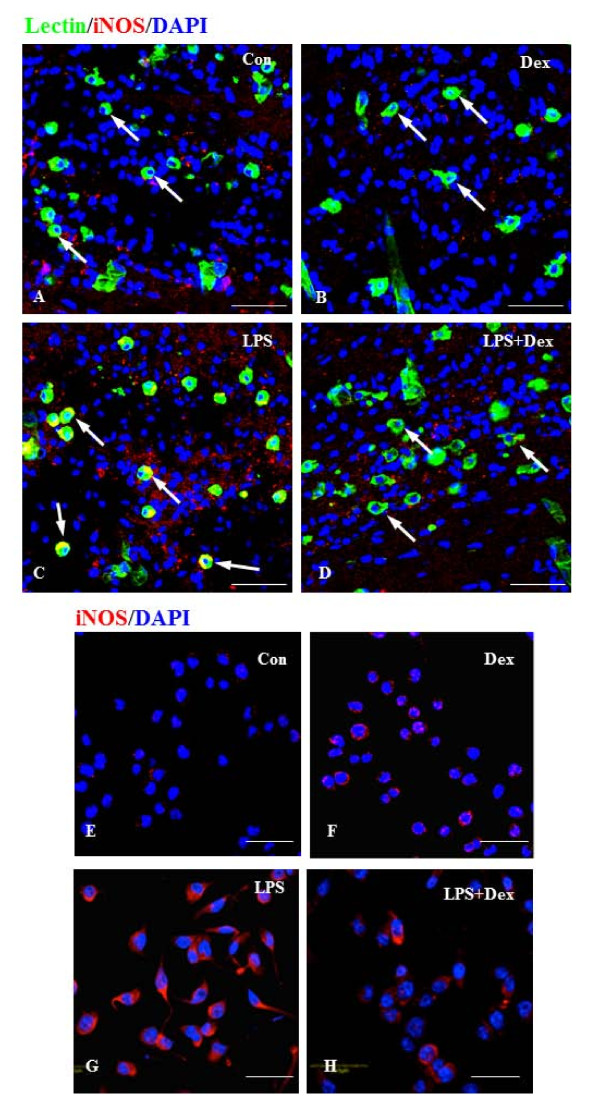
**A-H. Suppressive effect of Dex on iNOS expression in LPS-activated microglia in the corpus callosum of 3 day old rat brain and the BV-2 cells *in vitro***. Immunofluorescence analysis shows that iNOS (red) expression, which was hardly detectable in control cells (**A, E**) and cells treated with Dex alone (**B, F**). The induction of iNOS immunoexpression was evident in cells exposed to LPS (**C, G**). The induction was suppressed upon treatment with Dex (**D, H**). Note that the cells were counterstained with DAPI (blue). Scale bar (**A-H**); 50 μm

A major source of reactive nitrogen intermediates is inducible nitric oxide synthase (iNOS), an enzyme expressed in activated microglia during inflammation. Expression of iNOS protein was hardly detectable in microglia found in the corpus callosum of 3 day old rat brain (Figure. 4A) and the untreated BV-2 cells *in vitro *(Figure. 4E). There was marked induction of iNOS in the activated microglial cells *in vivo *and BV2 cells treated with LPS (Figure. 4C,G). The activated cells appeared to be hypertrophic and showed abundant cytoplasm that was intensely labeled with iNOS immunoreactivity, when compared with untreated cells. However, Dex (1.0 μM) treatment reduced the iNOS immunoreactivity in activated microglial and BV2 cells (Figure. 4D,H). Dex alone did not induce the expression of iNOS in microglial cells *in vivo *and BV2 cells *in vitro *(Figure. 4B,F).

**Figure 5:**
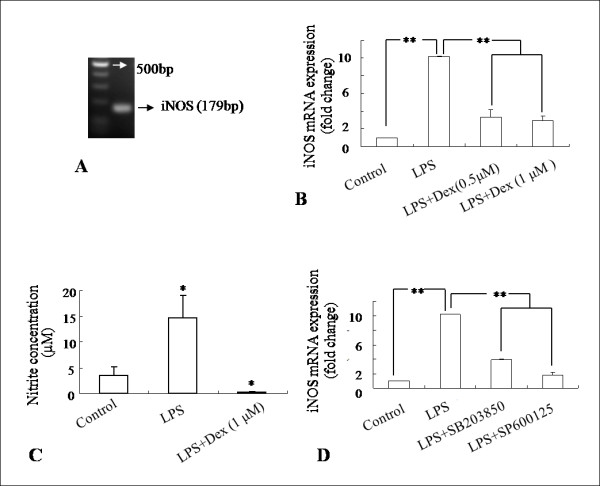
**A-D. Suppressive effect of Dex and inhibitors of p38 and JNK on iNOS expression and NO release in LPS activated BV-2 cells**. iNOS mRNA (179bp) was amplified (**A**) and effects of Dex and inhibitors of p38 and JNK on iNOS mRNA expression were examined by the real time RT-PCR. Both Dex (**B**) and inhibitors of p38 and JNK (**D**) decreased the induction of iNOS mRNA expression levels significantly in activated BV-2 cells when compared with that in LPS-treated cells. Nitrite assay further shows that NO release was inhibited by Dex in LPS-induced BV-2 cells (**C**). The data represent the mean ± SD (n = 3)* *p *< 0.05, ** *p *< 0.01.

Real time RT-PCR analysis showed that Dex (0.5 μM and 1 μM) decreased the induction of iNOS mRNA expression level in LPS-activated BV-2 cells (Figure. 5A,B) in which, the iNOS mRNA level was found to be elevated by about 10 folds in comparison to that of the untreated cells. There was no significant change in iNOS mRNA expression level in BV-2 cells treated with different concentrations of Dex alone, when compared to that in the control (data not shown). Nitrite assay revealed that the induction of NO release in activated BV-2 cells by LPS was attenuated significantly when the cells were exposed to Dex together with LPS (Figure. 5C). Interestingly, real time-RTPCR analysis showed that the iNOS mRNA expression level was decreased by pretreatment with inhibitors of p38 and JNK (SB203580 and SP600125, respectively) in activated BV-2 cells, suggesting that p38 and JNK MAPK pathways are involved in LPS-induced iNOS expression (Figure. 5D).

### Dexamethasone suppresses p38 and JNK MAPK pathways in BV-2 microglial cells treated with LPS

**Figure 6:**
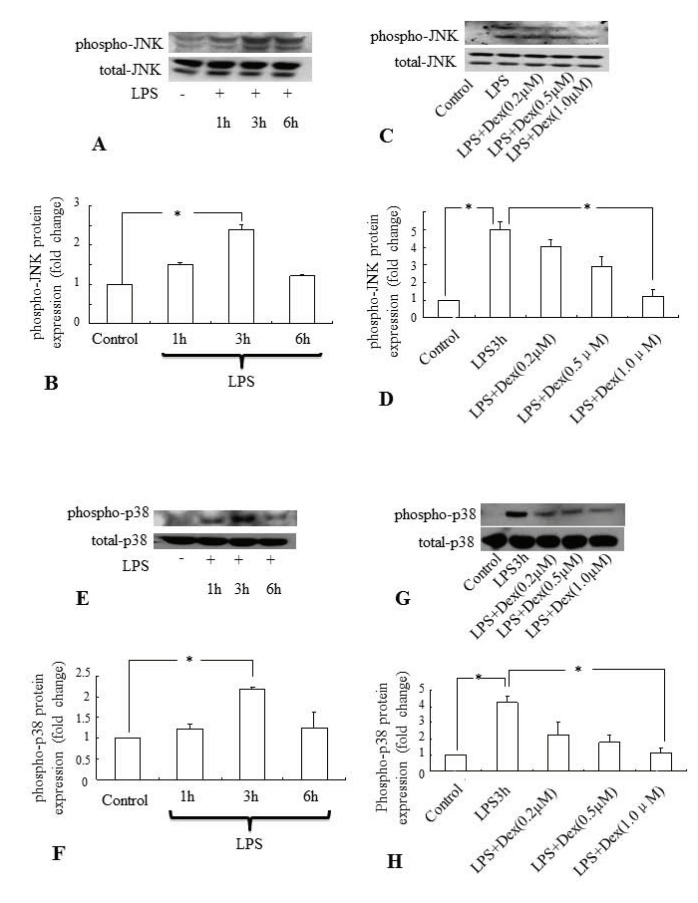
**A-H Quantitative western blot analysis showing the suppressive effect of Dex on the phospho-JNK and - p38 protein expression in LPS treated BV-2 cells**. Maximum induction of both phospho-JNK (**A,B**) and -p38 (**E,F**) protein expression was observed in microglia exposed to LPS for 3 h. The induction of both phospho-JNK (**C,D**) and -p38 (**G,H**) protein expression in microglia was found to be suppressed by Dex in a dose dependent manner. Panels **A, C, E **and **G **represent the Western blot of phospho-JNK and phospho-p38 protein of each experiment with corresponding quantitative analyses of total-p38 and -JNK proteins (Panels **B, D, F **and **H**), respectively. The data represent the mean ± SD (n = 3) * *p *< 0.05.

It has been previously shown that Dex inhibits the LPS-induced activation of MAP kinases in primary microglial cultures [26]. In this study, Western blot analysis showed that the LPS treatment at different time points induced the phosphorylation of JNK (Figure. 6A,B) and p38 (Figure. 6E,F) significantly in BV-2 cells. Maximum induction was observed at 3 h after LPS treatment. However, expression level of both phospho-p38 and phospho-JNK declined to the basal level at 6 h after incubation with LPS. The induction of phosphorylation of both JNK (Figure. 6C,D) and p38 (Figure. 6G,H) in BV-2 cells treated with LPS for 3 h was suppressed dose-dependently by the pretreatment of the cells with Dex.

### Dexamethasone suppresses Nox-2 expression *via *p38 and JNK MAPK pathways in BV-2 microglial cells treated with LPS

**Figure 7:**
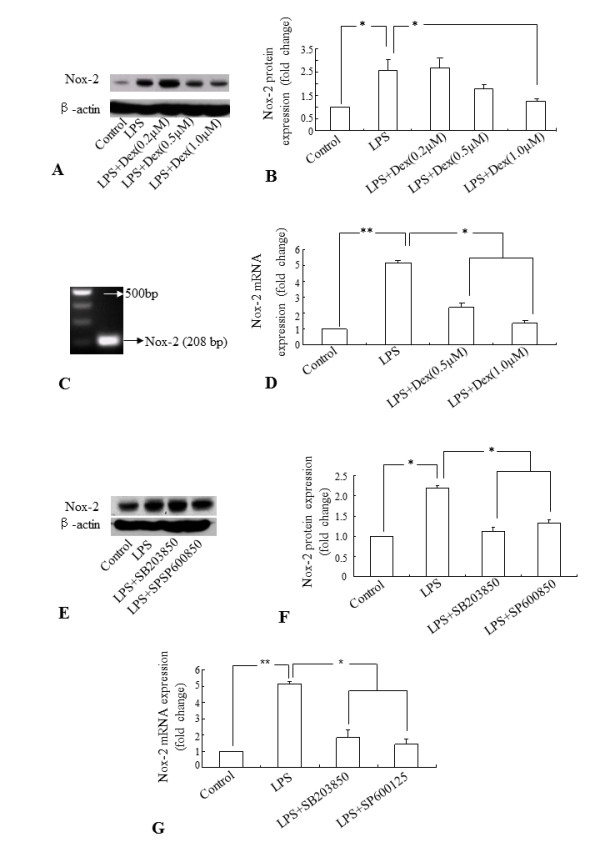
**A-G. Quantitative western blot and real-time RT-PCR analyses showing the suppressive effect of Dex and inhibitors of p38 and JNK on the induction of Nox-2 expression in LPS-treated BV-2 cells**. Panels **A **and **E **represent the Western blot of Nox-2 protein expression of each experiment with corresponding β-actin protein expression. Quantitative analysis of Western blot results are shown in panels **B **and **F**. The Nox-2 mRNA (208 bp) was amplified in microglia (**C**). The quantitative real time RT-PCR analysis shows that Dex (**D**) and inhibitors of p38 and JNK (**G**) suppressed the induction of Nox-2 mRNA expression in LPS-treated BV-2 cells, compared to that in cells treated with LPS. The data represent the mean ± SD (n = 3) * *p *< 0.05. ** *p *<0.01

Since Nox protein is considered to be critical in ROS production in activated microglia [10], we examined if Dex inhibits ROS production by acting on the Nox-2 expression in activated BV-2 cells. Incubation of LPS for 6 h significantly induced the Nox-2 protein expression level in BV-2 cells when compared with untreated cells (Figure. 7A,B). The LPS-induced Nox-2 expression was markedly suppressed by the addition of Dex (1 μM) in the culture and the inhibition was augmented with the increase in Dex concentrations (Figure. 7A,B). Real time RT-PCR results also showed that incubation of BV-2 cells with LPS for 6 h significantly induced Nox-2 mRNA expression and the increase was suppressed if the cells were pre-incubated with Dex (Figure. 7C,D). Next, we examined whether the inhibitory effect of Dex on Nox-2 is mediated *via *MAPKs pathways. Inhibitors of JNK and p38 showed a significant suppressive effect on Nox-2 protein expression in activated BV-2 cells (Figure. 7E,F). Real time RT-PCR also showed that mRNA expression of Nox-2 was significantly suppressed by inhibitors of JNK and p38 in LPS treated BV-2 cells (Figure. 7G).

### Knockdown of Nox-2 gene suppresses the LPS-induced ROS generation and NO release in BV2 microglial cells

**Figure 8:**
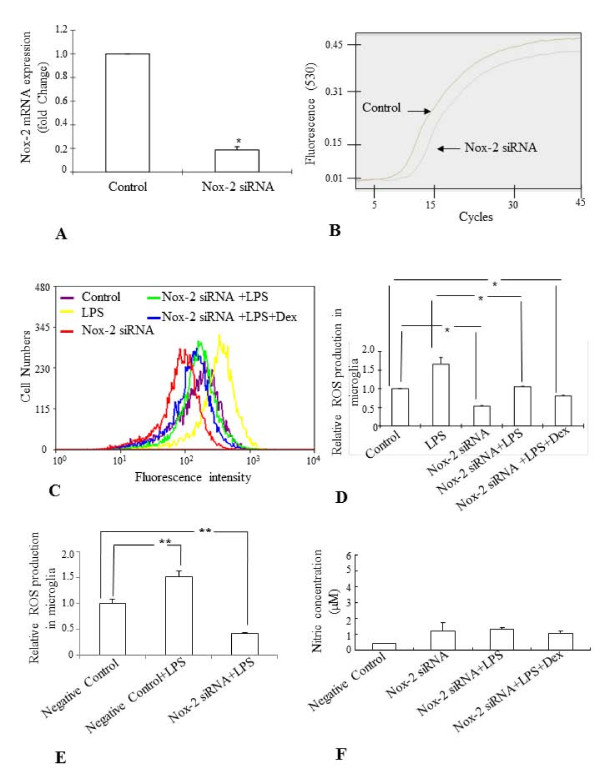
**A-F. Effect of knockdown of Nox-2 gene (Nox-2 siRNA) on the ROS production and NO release in BV-2 cells treated with LPS**. Real time RT-PCR quantitative analysis showed that about 75-80% of the endogenous Nox-2 gene expression was inhibited by its siRNA transfection in BV-2 cells (**A**). Panel **B **shows the Nox-2 mRNA amplification curve in Nox-2 siRNA treated and untreated BV-2 cells. The flow cytometric analysis shows that LPS did not induce ROS generation significantly in Nox-2 siRNA treated BV-2 cells compared to the LPS-treated non-transfected cells and preincubation of BV-2 cells with Dex further suppressed the induction of ROS production (**C, D**). Quantitative analysis of flow cytometric results are shown in panel D and E. Comparison between Nox-2 siRNA transfected cells and scrambled treated cells (Negative Control) also showed that LPS was unable to induce ROS production in the cells transfected with Nox-2 siRNA (**E**). Nitrite assay shows that LPS did not induce NO synthesis in Nox-2 siRNA treated BV-2 cells (**F**). The data represent the mean ± SD (n = 4) * *p *< 0.05; ** *p *< 0.01

Knockdown of endogenous Nox-2 in BV-2 cells (Nox-2 siRNA) by sequence specific siRNA (Figure. 8A,B) suppressed the ROS generation in control cells and prevented the LPS-induced ROS production in comparison to both control and scrambled siRNA (Negative Control) treated cells (Figure. 8C-E) and NO release (Figure. 8F).

### Overexpression of MKP-1 suppresses Nox-2 mRNA expression in BV-2 microglial cells

**Figure 9:**
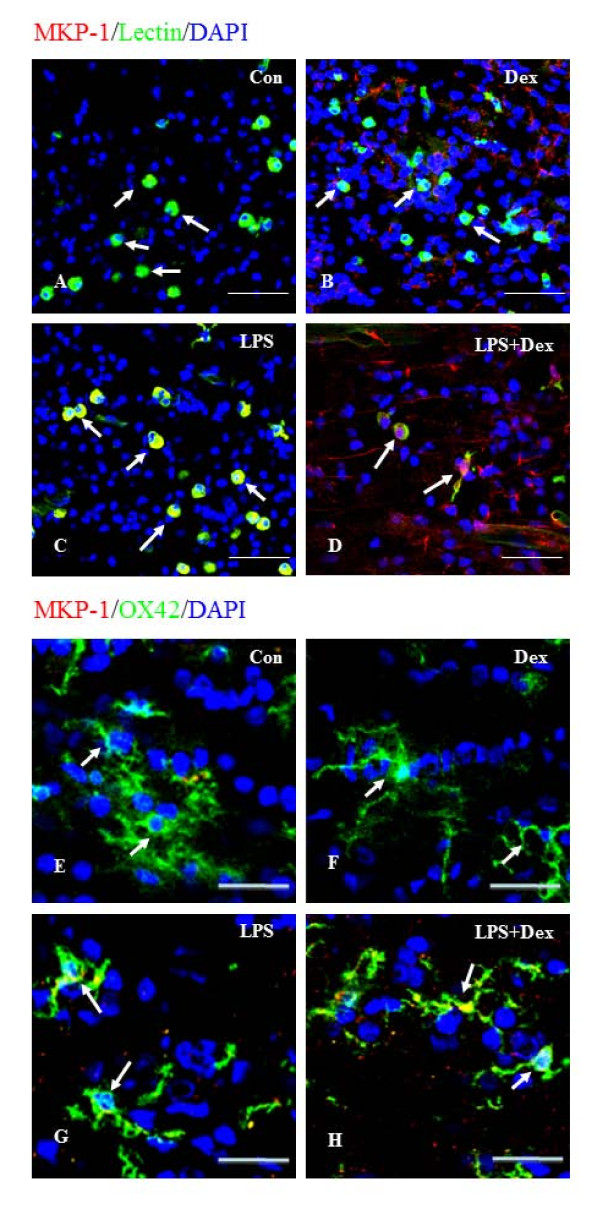
**A-H. Confocal images showing the MKP-1 expression (red) in lectin-positive (green) microglia in corpus callosum of 3 day postnatal and in OX42 postive (green) microglia in the corpus callosum of 4 week old rat brain subjected to different treatments**. MKP-1 immunoreactivity is hardly detectable in microglia of control rats injected with saline (**A, E**) and with Dex alone (**B,F**). MKP-1 immunoreactivity appears to be upregulated in microglia distributed in the corpus callosum region of rats that received LPS (**C, G**) or LPS and Dex (**D, H**).Scale bar (**A-D**), 50 μm; Scale bar (**E-H**), 20 μm.

**Figure 10:**
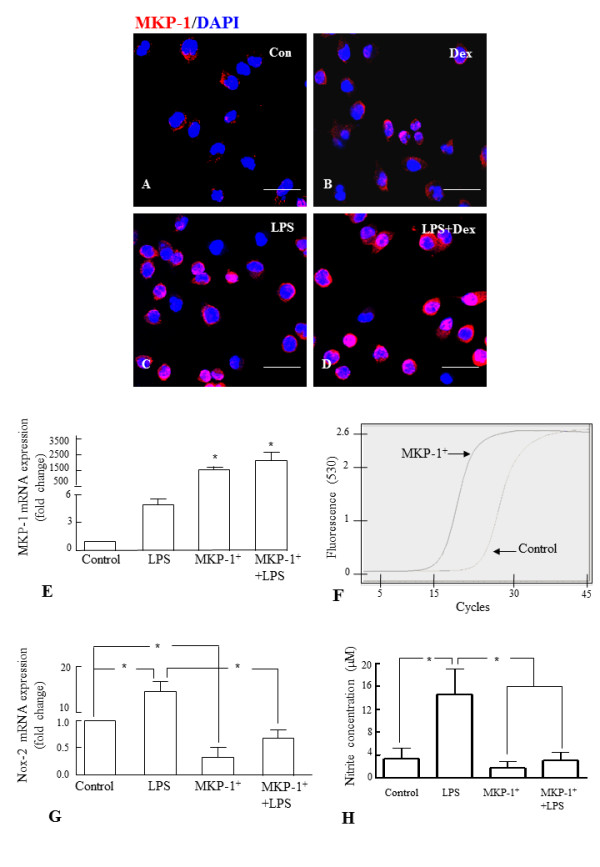
**A-H. Expression of MKP-1 in BV-2 cells and inhibition of Nox-2 expression and NO release in MKP-1-overexpressed BV-2 cells**. Immunofluorescence analysis shows that MKP-1 (red) expression, which was hardly detectable in control cells (**A**), and cells exposed to Dex alone (**B**), was induced in cells exposed to LPS for 6 h (**C**). The induction was further increased when the cells were pre-incubated with Dex (**D**). Note that the cells were counterstained with DAPI (blue). Panel E shows that MKP-1 mRNA expression fold change is significantly increased in BV-2 cells transfected with MKP (MKP-1^+^), compared with untreated cells (control). MKP-1 mRNA amplification curves for MKP-1^+ ^and control cells are shown in panel **F**. Furthermore, real time RT-PCR analysis and nitrite assay showed that MKP-1 overexpression significantly prevented the increase of Nox-2 mRNA expression level (**G**) and NO release (**H**) respectively, in MKP-1^+ ^BV-2 cells and MKP-1^+ ^cells treated with LPS. The data represent the mean ± SD (n = 3) * *p *< 0.05. Scale bar **(A-D)**, 50 μm

Since MAPKs pathways regulate Nox-2 expression and ROS production, we examined the role of MKP-1 in the regulation of Nox-2 by overexpressing MKP-1 in BV-2 cells. MKP-1 was localized in the microglia in the rat corpus callosum by immuofluorescence staining (Figure. 9A-H). In brain sections of normal 3-day old and 4 weeks old postnatal rats that received saline injection (Figure. 9A,E) or Dex injection (Figure. 9B,F), MKP-1 was hardly detectable in microglial cells distributed in the corpus callosum. However, microglia in the brains of rats that received LPS (Figure. 9C,G) or LPS plus Dex injection showed marked induction of MKP-1 immunoreactivity (Figure. 9D,H). A similar immunoexpression pattern of MKP-1 was observed in BV-2 cells (Figure. 10A-D). Real time RT-PCR results showed that MKP-1 mRNA expression level was significantly increased about 5 folds in LPS-treated BV-2 cells and about 2500 folds in MKP-1- transfected (MKP-1^+^) BV-2 cells when compared to that in untreated cells (Figure. 10E,F). In contrast, the mRNA expression level of Nox-2 was significantly reduced in MKP-1^+ ^BV-2 cells compared to that in untreated cells, and LPS treatment did not induce Nox-2 mRNA expression level in MKP-1^+ ^BV-2 cells (Figure. 10G). Moreover, nitrite assay also revealed that LPS did not induce the release of NO in MKP-1^+ ^BV-2 cells treated with LPS (Figure. 10H).

**Figure 11:**
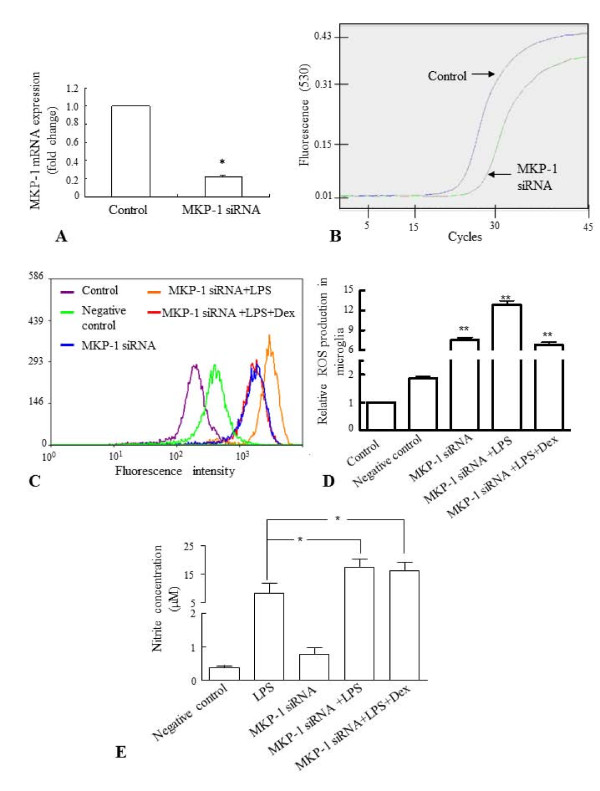
**A-E. Effects of knockdown of MKP-1 gene on ROS production and NO release in BV-2 cells treated with LPS**. Real time RT-PCR analysis showed that about 75-80% of the endogenous MKP-1 gene expression was inhibited in microglia after MKP-1 knockdown (MKP-1 siRNA) (**A**). Panel **B **shows MKP-1 mRNA amplification curves in MKP-1 siRNA treated cells and control cells. Panels **C **represents the results of flow cytometric analysis of DCF fluorescence intensity from one sample of each group obtained from three independent experiments performed. Inhibition of MKP-1 gene expression increased the ROS production in MKP-1 siRNA treated BV-2 cells as well as MKP-1 siRNA treated cells treated with LPS or LPS+Dex, compared with untreated BV-2 cells (control) or cells transfected with negative siRNA (negative control) (**C, D**). Similarly, inhibition of MKP-1 gene expression increased the NO release from LPS alone-treated cells as well as MKP-1 siRNA treated cells treated with LPS or LPS+Dex, compared to that from negative control BV-2 cells (**E**). The data represent the mean ± SD (n = 4), * *p *< 0.05; ** *p *< 0.01

On the other hand, knockdown of MKP-1 (MKP-1 siRNA) with sequence specific siRNA (Figure. 11A,B) significantly increased the ROS production (Figure. 11C,D) and NO release (Figure. 11E) by BV-2 cells exposed to LPS with or without Dex, in comparison to that of controls.

## Discussion

Activated microglial cells by neurodegenerative stimulus undergo a defined pattern of physiological changes and release a number of proinflammatory cytokines (such as IL-1, IL-6 and TNF-α) and chemokines (MCP-1) that contribute to neuropathogenesis in CNS inflammation [3,26]. In addition, these cells release free radicals such as superoxide, ROS and NO. Excessive production of ROS and reactive nitrogen species (RNS) by activated microglia, has the potential to harm neighboring healthy cells (neurons and other glial cells) and is the main cause of oxidative stress in the nervous system, leading to neurodegenerative disorders such as Alzheimer's disease [15,17]. Therefore, suppression of microglia-mediated neuroinflammation and neurotoxicity has been considered as an important strategy in neurodegenerative disease therapy. Several drugs have been shown to alleviate symptoms of neurodegenerative diseases by suppressing the inflammatory response of microglial cells. However, chronic use of these drugs is often associated with debilitating side effects, and none seems to restrain the progression of these diseases. It is suggested that downstream targets of these drugs may limit the side effects of those drugs in clinical setting.

Dexamethasone, a synthetic immunosuppressor has been shown to suppress ROS production, NO release as well as inflammatory reaction of activated microglial cells [20,21]. However, the mechanisms by which Dex suppresses ROS production and NO release remained unclear. The present study showed that the increased ROS production and NO release in activated microglia were associated with increased expression of Nox-2 (also known as gp91*phox*) and iNOS, which are the major sources of ROS and reactive nitrogen intermediates respectively, in activated microglia. The expression of Nox-2 has been localized in neurons and glial cells including microglia [10] and its upregulation and subsequent ROS production in activated microglia were found to be suppressed by Dex. Moreover, Dex has been shown to provide neuroprotection by inhibiting iNOS expression as well as NO synthesis in activated microglia [27,28]. Hence it is suggested that Dex suppresses the production of ROS and NO release, and subsequently neuronal oxidative damage by inhibiting the expression of Nox-2 as well as iNOS in activated microglia. The inhibition of Nox-2 by Dex observed in activated microglia appears to be mediated by MKP-1 dependent suppression of MAPKs, since the overexpression of MKP-1 and inhibition of MAPKs downregulate the expression of Nox-2 in activated microglia.

Nox-dependent ROS generation has been shown to induce the expression of TNF-α [12], TGF-β1, and MCP-1 through MAP kinase activation [11] or through transcription factors, including NFκB, AP-1,and p53, which contain redox-sensitive, low-p*K*a cysteine residues in their DNA binding domain [29-31]. The Nox-dependent ROS generation by microglia has also been implicated in a variety of CNS diseases including stroke [32], ischemic brain injury [33], demyelinating disease [34] and inflammatory neurodegeneration, including Alzheimer's disease (AD) and Parkinson's disease [35]. Hence, suppression of NOX-dependent ROS generation may form a better therapeutic strategy for neuroinflammation and neurodegenerative diseases.

Microglial response to extracellular stimuli is mediated by kinase and phosphatase cascades. Several reports have demonstrated that p38 and p44/42 families of MAPK pathways play a significant role in activation of microglial cells which in turn leads to release of neurotoxic molecules and neuroinflammation in acute brain injury such as stroke and in chronic neurodegenerative diseases such as AD [24,36,37]. Recently, we have demonstrated that JNK and p38 MAPKs in activated microglia contribute to the induction of MCP-1, a chemokine which promotes the migration of microglia leading to amplification of the inflammation process in the injury site [26,38]. Glucocorticoids (GC) or Dex have been consistently shown to inhibit the expression of proinflammatory genes by antagonizing the MAPK pathways, in particular the p38 and JNK pathways. The inhibition of MAPK pathways is mediated via induction of MKP-1 [26,39-42]. Several proinflammatory stimuli, such as LPS and neuromodulators such as endocannabinoid anandamide also induce MKP-1 expression which, however, activates a negative feedback loop down-regulating the production of pro-inflammatory cytokines, chemokines and neurotoxic molecules such as TNF-α, IL-1 and IL-6, MCP-1 and NO [26,39,43,44]. The induction of MKP-1 has been shown to be a survival mechanism against oxidative damage in cancer cell lines [45]. Our study supports the notion that Dex inhibits JNK and p38 phosphorylation in microglial cells by inducing MKP-1 expression, which in turn negatively regulates the proinflammatory response and chemotactic effect of activated microglia [26,39].

More recently, ROS has been shown to inhibit MKP-1 activation causing activation of JNK, which contributes to pancreatic beta-cell death [46] and to function as an upstream regulator of JNK/SAPK and p38 MAPK [47]. On the contrary, MKP-1 activation by Dex in activated microglia appears to limit the oxidative damage by suppressing Nox-dependent ROS production. Interestingly, overexpression and siRNA analyses of MKP-1 clearly reveal that inhibition of Nox-dependent ROS production by Dex in activated microglia is mediated *via *upregulation of MKP-1, indicating a complex link between ROS production and MKP-1 phosphorylation in activated microglia. Moreover, this paradox could be due to the different metabolic mechanisms in different cell types.

## Conclusion

Overall, the overexpression of MKP-1 in microglia may be considered as the possible therapeutic option as it suppresses the ROS-mediated neurotoxicity caused by activated microglia in neurodegenerative diseases. However, further studies involving appropriate animal models are required to determine MKP1 as the possible therapeutic target, since the influence of other microenvironmental factors on microglial activation cannot be excluded.

## Methods

### Cell Culture

BV-2 cells (widely used murine microglial cell line) were maintained at 75 cm^2 ^culture flasks in Dulbecco's Modified Eagle's Medium (DMEM, Sigma, St. Louis, MO, USA; Cat. No. 1152) supplemented with 10% fetal bovine serum (FBS, HyClone, Logan, UT) and 1% antibiotic antimycotic solution (Sigma, St. Louis, MO, USA; Cat. No. A5955), and cultured in 37°C in a humidified atmosphere of 5% CO_2 _and 95% air incubator. Cells were plated on 96-well plates at about a density of 1.0 × 10^6 ^per 100 mm^2 ^culture dish for flow cytometric assay and RNA isolation, at 2.0 × 10^5 ^per well on a 24-well plate for immunocytochemistry and at 1.0 × 10^6 ^per well on a 6-well plate for gene silencing MKP-1 transfection studies. On the following day after plating, cells were subjected to different treatments.

### Treatment of the BV-2 microglial cell culture

The dose- and time-dependent effects of LPS on ROS production in BV-2 cells have been carried out to determine the optimal effective concentration and exposure time of LPS. BV-2 cells were incubated in medium containing LPS (Sigma-Aldrich,St. Louis, MO, USA; Cat. NO. L2762) at various concentrations (0.05, 0.1, 0.3, 1.0 and 1.5 μg/ml) for 6 h. Subsequently, BV-2 cells were treated with LPS at 1 μg/ml for 2, 4, 6, 12 and 24 h to establish the time-dependent effects of LPS.

In order to study the effect of dexamethasone (Dex) on the ROS production in LPS activated BV-2 cells, the cells were washed with 0.1 M phosphate buffered saline (PBS), followed by incubation in medium containing 1 μg/ml LPS (Sigma-Aldrich,St. Louis, MO, USA; Cat. NO. L2762) for 6 h, with or without a 30 min pre-incubation of Dex (Sigma-Aldrich, St. Louis, MO, USA; Cat. No.D2915) at various concentrations (0.2 μM, 0.5 μM and 1.0 μM). To ascertain the involvement of MAPK pathway in ROS production and iNOS mRNA expression, BV-2 cells were incubated with either SP600125 (1 μM, A.G.. Scientific Inc., St. Diego, CA, USA; Cat. No. S2022) or SB203580 (1 μM, Merck, Darmstadt, Germany; Cat. No.559389), inhibitors of JNK and p38 respectively, for 30 min prior to LPS treatment for 6 h.

### Measurement of ROS production in BV-2 microglia by Flow cytometry

Intracellular ROS production was determined by detecting the fluorescent intensity of 2', 7'-dichlorofluorescein (DCF), the oxidized product of the fluoroprobe 5-(and 6)-chloromethyl-2', 7'-dichlorodihydrofluorescein diacetate (CM-H_2_DCFDA, Molecular Probers, USA; Cat. No. C6827). Briefly, BV-2 cells grown in 100 mm^2 ^culture dish at the density of 1.0 × 10^6 ^were treated as described above. Subsequently, the cells were trypsinized into dissociated cells and collected by centrifugation into a 15 ml Falcon tube. The dissociated cells were re-suspended in 0.5 ml of pre-warmed PBS containing CM-H_2_DCFDA (10 μmol/l) and allowed to recover for 40 min in the darkness. After the incubation, the cells were immediately measured with a flow cytometer with excitation at 488 nm and emission at 535 nm (CyAn ADP, DakoCytomation, USA) and the data obtained was analyzed with Summit V4.2 software. The amount of ROS production was considered to be directly proportional to the fluorescence intensity.

### RNA isolation and Real-time RT-PCR

Total RNA from BV2 cells was extracted with RNeasy Mini Kit (Qiagen, Germany, Cat. No. 75161) according to the manufacturer's instructions and quantified spectrophotometrically. 2 μg of RNA from each sample was added to a total volume of 25 μl reaction mixture containing 2.5 μM of oligo (dT) primer (Promega, Madison, WI USA; Cat. No. C110A), and 200U of Molony Murine Leukemia Virus Reverse Transcriptase (M-MLV, Promega, Madison, WI, USA; Cat. No. M5314). The reaction was initiated by incubating the reaction mixture for 1 h at 42°C for reverse transcription, and stopped by heating for 10 min at 70°C. Aliquot (0.5 μl) of the each reverse transcription product was added to the 20 μl reaction mixture containing LightCycler-FastStart DNA Master SYBR Green І, 0.5 μM of each primer corresponding to mouse iNOS, Nox-2, MKP-1 or β-actin, and 4 mM MgCl_2 _to amplify the genes in a LightCycler (Roche, Germany). The primer sequences used in this study are listed in Table 1. After pre-incubation at 95°C for 10 min, the polymerase chain reaction (PCR) was performed as follows: 35 cycles of denaturation at 95°C for 15s, annealing at 60°C for 5s, and elongation at 72°C for 12s. 2^-[ΔΔCt] ^method was used to compare the mRNA expression levels of genes in experimental groups with control groups [48].

**Table 1:** Gene names and primer sequences used for real time RT-PCR

Gene	Primers
Β- actin	5'-tcacccacactgtgcccatctacga-3' 5'-ggatgccacaggattccataccca-3'

iNOS	5'-cgtgtgcctgctgccttcctgctgt-3' 5'-gtaatcctcaacctgctcctcactc-3'

Nox-2	5'-ccaactgggataacgagttca-3' 5'-gagagtttcagccaaggcttc-3'

MKP-1	5'-tggagatcctgtccttcctg-3' 5'-gtctgccttgtccttgtcct-3'

### Immunofluorescence staining in BV-2 microglial cells

BV-2 cells were plated onto poly-L-lysine coated cover slips in 24-well plate. For immunofluorescece, the cells subjected to different treatments were fixed with 4% paraformaldehyde at room temperature for 30 min, washed with PBS for 30 min and blocked with 1.0% bovine serum albumin (BSA) for 30 min. The cells were then incubated overnight with rabbit-anti-iNOS (1:400; Chemicon, Temecula, CA, USA; Cat. No. AB5382) or purified mouse anti-gp91phox (1:500; BD Biosciences, San Jose, CA, USA; Cat. No. 611414). Subsequently the cells were incubated with Cy3-gonjugated goat anti-rabbit or goat anti-mouse secondary antibodies (1:200, Chemicon International, Temecula, CA, USA; Cat. No. AP132C; Cat. No. AP124C), counter-stained with DAPI and examined under a confocal microscope (Fluoview 1000, Olympus, Tokyo, Japan)

### Double immunofluorescence staining on postnatal rat brain sections

Three day-old and four week-old Wistar rats were purchased from the Laboratory Animal Centre, National University of Singapore. In the handling and care of animals, the International Guiding Principles for Animals Research, as adopted by the Institutional Animal Care and Use Committee, National University of Singapore, were followed. All efforts were made to minimize pain and the number of rats used. Two injections of LPS (1 mg/kg diluted in 250 μl of saline per injection) were given to the pups with an interval of 6 h. 12 h after the second injection, the pups were perfused and fixed with 4% paraformaldehyde for further procedure. In control group, LPS injections were replaced by two injections of saline (250 μl per injection). A single dose of Dex (1 μmol diluted in 250 μl saline) for 3 day-old pups and a single dose of Dex (10 μM diluted in 250 μl saline) for 4 week-old rats was given 6 h after the second injection of LPS. Dex was allowed to function for 6 h before the pups were sacrificed for further procedure. For double immunofluorescence staining, rat brains were cut through the corpus callosum. All sections were incubated with purified mouse anti-gp91phox Ig (1:250; Cat No. 611414, BD Biosciences Pharmingen, Heidelberg, Germany) or rabbit anti-MKP-1 (1:250; Cat No. sc-1102, Santa Cruz Biotechnology, Inc. CA, USA) or mouse anti-iNOS (1:500, Cat No. 610431, BD Biosciences Pharmingen, Heidelberg, Germany) overnight at room temperature. Only the tissue sections cut through the corpus callosum of 4 weeks old rat brain were additionally incubated with mouse anti-OX-42 (1:50; Cat No. CBL1512, Millipore, MA, USA). On the following day, the sections were further incubated with Cy3-conjugated goat-anti-mouse secondary antibody (1:200; Chemicon, Temecula, CA, USA) or Cy3-conjugated goat-anti-rabbit secondary antibody (1:200; Cat No. AP132C, Chemicon) and tomato (*Lycopersicon esculentum*) lectin (1:800; Sigma-Aldrich, St. Louis, MO, USA; Cat. No. L0401) or FITC-conjugated goat-anti-mouse IgG (1:100; Cat No. F9137, Sigma-Aldrich Co., MO, USA) for 1 h. The sections were counterstained with DAPI (1 μg/ml, Invitrogen, USA; Cat. No. D1306) and mounted with fluorescent mounting medium (Dako Cytomation, Glostrop, Denmark). Photo-images were captured using a confocal microscope (Olympus FV1000, Tokyo, Japan).

### Western blot assay

Total protein of BV-2 cells exposed to different treatments was extracted using the Protein Extraction Kit (Thermo Fish Scientific Inc., Rockford, IL, USA; Prod#78501) and HaltTM Protease Inhibitor Cocktail Kit (Thermo Fish Scientific Inc., Rockford, IL, USA; Prod#78410). The protein level was quantified using a protein assay kit (Bio-Rad, Hercules, CA, USA; Cat. No.500-0007). 20 μg of protein extracts were separated on 10% SDS-polyacrylamide gels and transferred to polyvinylidene difluoride transfer membranes. The membranes were blocked with 5% non-fat dry milk and incubated with primary antibodies over night at 4°C. The primary antibodies used are as follows: rabbit anti-phospho-JNK, total JNK, phosphor-p38 and total p38 (1:1000, all from Cell Signaling Technology, Beverly, MA, USA; Cat. No. 9250, and 9210), gp91phox (1:1000, BD Biosciences, San Jose, CA, USA; Cat. No. 611414) and mouse anti-β-actin monoclonal antibody (Sigma-Aldrich, St. Louis, MO, USA; A1978)

The next day, the membranes were incubated with the horseradish peroxidase-conjugated secondary antibody (Cell Signaling Technology, USA; Cat. No. 7074) for 1 h. The immunoblots were developed with the enhanced chemiluminescence detection system. The signal intensity was measured with Quantity One Software (Bio-rad, Hercules, CA, USA) version 4.4.1.

### Knockdown of Nox-2 and MKP-1 by siRNA in BV-2 microglial cells

MKP-1 and Nox-2 expression in BV-2 cells was inhibited separately using siRNA (Silencer siRNA transfection II kit, Cat No. 1631; Ambion, Inc. USA). Pre-designed siRNA for MKP-1 (sense:GAGGGAGAGUGUUUGUUCAtt; antisense:UGAACAAACACUCUCCCUCca) and Nox-2 (sense:GGUCUUAUUUUGAAGUGUUtt; antisense:AACACUUCAAAAUAAGACCtc) (Refer Table 2 for all the siRNA sequences of MKP-1 and Nox-2) and negative control siRNA were obtained (Ambion, Inc. USA). The siRNA transfection in BV-2 cells was performed according to the manufacturer's instruction. Transfection complexes were prepared by mixing oligofectamine (Ambion, Carlsbad CA USA, Cat. No.12252-011) and siRNAs (100 nM) in Opti-Mem Medium (Invitrogen, Life Technology, USA). The cells were then mixed with transfection complexes and incubated for 24 h in 6-well (2x10^5 ^cells/well). Subsequently, the culture of both MKP-1/Nox-2 siRNA and negative control siRNA transfected BV-2 cells were exposed to LPS with or without Dex. The transfection efficiency was assessed by quantitative real time RT-PCR using specific primers (Table 1) for MKP-1 and Nox-2 genes.

**Table 2:** siRNA construct sequences used in the study for knockdown experiments

Gene	siRNA sequence
MKP-1 (ID: s72445)	Sense: GGUUCAACGAGGCUAUUGAtt Antisense: UCAAUAGCCUCGUUGAACCag

MKP-1 (ID: s72446)	Sense: GAGGGAGAGUGUUUGUUCAtt Antisense: UGAACAAACACUCUCCCUCca

MKP-1 (ID: s72447)	Sense: GGUCACUACCAGUACAAGAtt Antisense: UCUUGUACUGGUAGUGACCct

Nox-2 (ID: s64649)	Sense: GCAUAAUUUAGAUAUCUGUtt Antisense: ACAGAUAUCUAAAUUAUGCtc

Nox-2 (ID: s64650)	Sense: GGUCUUAUUUUGAAGUGUUtt Antisense: AACACUUCAAAAUAAGACCtc

Nox-2 (ID: s64651)	Sense: CGGUGACAAUGAGAACGAAtt Antisense: UUCGUUCUCAUUGUCACCGat

The sequences highlighted in the bold were used in this study.

### MKP-1 overexpression in BV-2 microglia

The transfection of MKP-1 (Addgene plasmid 13469: pWay21-MKP-1 FL) [49] into BV-2 cells was performed using transfection reagent (LipofectamineTM 2000, Invitrogene, Carlsbad, CA, USA; Cat. No. 116688-027), according to manufacturer's instruction. BV-2 cells were incubated in 10% FBS-DMEM without antibiotics for 24 h to reach 90-95% confluency. The plasmid (4 μg) was mixed with 12 μl of transfection reagent and incubated for 20 min at room temperature. The cells were incubated in 2 ml OptiMEM Reduced Serum Medium with the above transfection mixture for 6 h. Then the cells were incubated in 10% FBS-DMEM for 24 h and subjected for various treatments. Some cells were transfected with vector backbone alone without the MKP-1 insert to assess the transfection efficiency (data not shown).

### Statistical analysis

The data were presented as mean ± SD. Statistical significance was evaluated by either the Student's test or one-way ANOVA analysis of variance. Results were considered as significant at *p *< 0.05.

## Abbreviations

CM-H2DCFDA: 5-(and 6)-chloromethyl-2', 7'-dichlorodihydrofluorescein diacetate CNS, central nervous system; DAPI, 4':6-diamidino-2-phenylindole; DCF, 2': 7'-dichlorofluorescein; Dex: dexamethasone; DMEM: Dulbecco's modified Eagle's medium; FBS: fetal bovine serum; FITC: fluorescein isothiocyanate; GC: glucocorticoids; IL-1β: interleukin-1β; iNOS: iducible nitric oxide synthase; JNK: c-Jun N-terminal kinase; LPS: lipopolysaccharide; MAPK: mitogen-activated protein kinase; MCP-1: monocyte chemoattractant protein 1; MKP-1: MAPK phosphatase-1; NADPH: Nicotinamide adenine dinuceotide phosphate; NO: nitric oxide; RNS: reactive nitrogen species; ROS: reactive oxygen species; RT-PCR: reverse transcriptase polymerase chain reaction; TNF-α: tumor necrosis factor-α

## Competing interests

The authors declare that they have no competing interests.

## Authors' contributions

YH performed majority of the experiments and wrote the manuscript. PR performed some experiments and participated in revising the manuscript. EAL helped in the design of the experiments and participated actively in discussion of the project and editorial work of the manuscript. STD is the Principal Investigator and was instrumental to the execution of the entire project. All of the authors have read and approved the final version of the manuscript.
